# Analysis of the InsCor Score as a Predictor of Mortality in Patients Undergoing Coronary Artery Bypass Grafting

**DOI:** 10.21470/1678-9741-2020-0339

**Published:** 2021

**Authors:** Iuri Ferreira Félix, Nilzo Augusto Mendes Ribeiro, Valcellos José da Cruz Viana, Adriana Lopes Latado

**Affiliations:** 1 Department of Internal Medicine and Diagnosis Support, Escola de Medicina, Universidade Federal da Bahia, Salvador, Bahia, Brazil.; 2 Department of Cardiac Surgery, Hospital Santa Izabel, Salvador, Bahia, Brazil.

**Keywords:** Risk Measurement, Hospital Mortality, Cardiac Surgical Procedures, Myocardial Revascularization, Surgeons

## Abstract

**Introduction:**

Risk scores are important tools for predicting adverse events in cardiac surgery, but their accuracy varies when applied to different populations. The objective of this study is to evaluate the performance of the Brazilian score InsCor as a predictor of mortality after coronary artery bypass grafting (CABG) compared to the European System for Cardiac Operative Risk Evaluation (EuroSCORE) and Society of Thoracic Surgeons (STS) scores.

**Methods:**

This is an observational and retrospective study, with patients undergoing surgical myocardial revascularization in a cardiology hospital in Salvador (Bahia, Brazil), between 2010 and 2015. InsCor, STS, and EuroSCORE were compared for accuracy in predicting mortality within 30 days after surgery. Discrimination capacity of models was assessed using areas under receiver operating characteristic (ROC) curves. Significance level was 5%.

**Results:**

Four hundred sixty-one patients were evaluated (mean age 63 [± 8.6] years, 77% men). Thirty-day mortality was 2.6%. InsCor classified 88, 210, and 163 patients as having low, medium, and high risk of death, respectively. According to EuroSCORE and STS, 379 and 430 patients were classified as having low risk and 77 and 29 as medium risk, respectively. Area under the ROC curve was 0.734 (*P*=0.002) for InsCor, 0.615 (*P*=0.027) for EuroSCORE, and 0.623 (*P*=0.033) for STS. ROC curve of InsCor maintained statistical significance after adjustment for other models.

**Conclusion:**

The InsCor score, derived from a Brazilian sample, showed good predictive accuracy of death up to 30 days in patients undergoing CABG in relation to STS and EuroSCORE scores.

**Table:** 

Abbreviations, acronyms & symbols				
AUC	= Area under the ROC curve		IIQ	= Interquartile range
BMI	= Body mass index		LMC	= Left main coronary
CABG	= Coronary artery bypass grafting		LV	= Left ventricular
CI	= Confidence interval		LVEF	= Left ventricular ejection fraction
COPD	= Chronic obstructive pulmonary disease		MI	= Myocardial infarction
CPB	= Cardiopulmonary bypass		PAD	= Peripheral arterial disease
DATASUS	= Brazilian National Health System Information Technology Department		PCI	= Percutaneous coronary angioplasty
			ROC	= Receiver operating characteristic
EuroSCORE	= European System for Cardiac Operative Risk Evaluation		SAH	= Systemic arterial hypertension
			SD	= Standard deviation
ICU	= Intensive care unit		STS	= Society of Thoracic Surgeons

## INTRODUCTION

Cardiovascular diseases, especially coronary artery diseases, are a worldwide and Brazilian public health problem due to their high morbidity and mortality. The estimated prevalence of angina is 12-14% in men and 10-12% in women, and approximately 400,000 new cases of acute myocardial infarction occur annually in the Brazilian population ^[^^1^^]^.

According to the Brazilian National Health System Information Technology Department (DATASUS), cardiovascular diseases account for up to 30% of the causes of death in the country, with coronary artery disease and stroke being the most common causes ^[^^1^^]^. The use of scores to predict adverse events during and after medical procedures is an objective way of analyzing the perioperative risk, allowing the implementation of better directives ^[^^2^^-^^4^^]^. However, these indicators can present heterogeneous performances according to the risk profile of different populations, which tends to limit their use ^[^^5^^-^^7^^]^.

In cardiac surgery, the most common national and international preoperative risk scores are the European System for Cardiac Operative Risk Evaluation (EuroSCORE) and the Society of Thoracic Surgeons (STS) scores. The first was created from a European database and revised in 2010, being renamed EuroSCORE II ^[^^8^^]^. The second was designed from a database of patients from the United States of America who underwent coronary artery bypass grafting (CABG) ^[^^9^^]^. Both scores classify patients at different risk levels (high, medium, or low) for the incidence of undesirable perioperative outcomes, fatal and non-fatal, during hospitalization or in a long postoperative period, up to 30 days.

The InsCor score was created by a surgical team from the Universidade de São Paulo (São Paulo, Brazil) in 2012, based on a national observational and prospective study, and aimed to adapt the analysis to our reality. The authors showed that InsCor was useful for predicting cardiovascular risk in the selected sample, with the potential for a superior performance to some older models ^[^^4^^]^. However, this model has still been poorly evaluated in our population.

Considering this, the present study aims to analyze the performance of InsCor in predicting the risk of mortality in patients undergoing myocardial revascularization, in comparison with the international models most used in our country.

## METHODS

### Design and Sampling

This is a retrospective cohort study, in which records from the database of the surgical team of a cardiology reference hospital in Salvador (Bahia, Brazil) were analyzed. Four hundred sixty-one adult patients were admitted consecutively to undergo CABG between October 2010 and April 2015, with a minimum post-surgical follow-up time of 30 days. Exclusion criteria were as follows: patients with incomplete or absent information about postoperative hospital complications in the database; and patients undergoing surgical procedures associated with CABG, such as valve replacement/repair or correction of intracardiac shunts.

### Data Collection and Variables of Interest

The research information was collected from a hospital database and the variables of interest were:

### 1) Independent Variables

The selection of independent variables was based on the predictive models developed and validated in studies previously published in the literature. The EuroSCORE and STS scores were the risk prediction models used as reference. The national model chosen for validation in the study sample was the InsCor score.

The clinical and demographic variables, and their respective units or definitions, were: sex; age, in years; weight, in kilograms; height, in meters; body mass index, defined by the ratio of weight in kilograms to the square of height in meters; chronic obstructive pulmonary disease (medical diagnosis and use of dilator or inhaled corticosteroids); non-coronary obstructive arterial disease (medical diagnosis of peripheral arterial obstructive disease and/or obstruction of carotid arteries > 50%); left ventricular systolic function (normal, if left ventricular ejection fraction [LVEF] > 50%, mild/moderate dysfunction, if LVEF is between 30-50%, and severe dysfunction, if LVEF < 30%); previous neurological dysfunction (motor dysfunction affecting walking or daily functions); previous cardiac surgery; serum creatinine levels (pre and postoperative); unstable angina (using intravenous nitrate); recent myocardial infarction (up to 90 days); moderate/severe pulmonary arterial hypertension (pulmonary artery systolic pressure > 60 mmHg); post-infarction interventricular communication; diabetes mellitus (using an oral hypoglycemic agent or insulin); smoking (current or not); systemic arterial hypertension (current antihypertensive use); dyslipidemia (total cholesterol > 200 mg/dl, triglycerides > 150 mg/dl, high-density lipoprotein cholesterol < 50 mg/dl for women and < 40 mg/dl for men); total number of coronary stenosis over 75%; left coronary trunk injury > 50%; and preoperative hypoxemia (arterial oxygen pressure < 60 mmHg).

The variables related to the cardiac disorder were emergency/urgent surgery (need for intervention < 48 hours, due to imminent risk of death or unstable clinical and hemodynamic state) and hemodynamic instability (ventricular tachycardia, ventricular fibrillation, post-cardiac arrest, mechanical ventilation, or use of an intra-aortic balloon).

### 2) Dependent Variables

The primary outcome of the study was death within 30 days after myocardial revascularization, including deaths from all causes. Other outcomes defined as major morbidities were also evaluated: stroke (diagnostic imaging plus central neurological deficit persisting for > 72 hours); prolonged endotracheal intubation (> 48 hours); reoperation (due to tamponade or the need for hemostasis); and mediastinitis (need for surgical reintervention and use of antibiotic therapy, with or without a positive culture).

Myocardial revascularization was considered complete when all coronary obstructions planned for treatment, in the preoperative period, were addressed.

### Statistical Analysis

Statistical analysis was performed using R software (R core Team, Vienna, Austria) for Windows and MedCalc, version 18.5 for Windows. Descriptive statistics were performed, with categorical variables described as proportions and quantitative variables described as means (standard deviation) and medians (interquartile range), according to the normality of the data. Quantitative variables were assessed for normality using Shapiro-Wilk statistical test and distribution characteristics (asymmetry and kurtosis). The discriminating power of the scores was analyzed using the C statistic (receiver operating characteristic [ROC] curve), in which the areas under the ROC curve were compared between InsCor, STS, and EuroSCORE risk scores. Multivariate analysis in the model of unconditional logistic regression was used to test the association between the level of risk of death predicted by a score and the patient’s mortality, adjusted for the other scores. The calibration of the models was evaluated by the Hosmer-Lemeshow test. For the purposes of statistical inference, *P*-value < 0.05 was considered statistically significant.

### Ethical Aspects

The present study complied with the ethical principles that involve research on human beings, as guided by resolution no. 466/2012, of the National Health Council. The project was evaluated and approved by the Research Ethics Committee of the Faculdade de Medicina da Bahia, Universidade Federal da Bahia, opinion n^o^ 2,709,313 and CAAE n^o^ 90426818.2.0000.5577 of June 13, 2018, and by the Research Ethics Committee of Hospital Santa Izabel, opinion n^o^ 2,759,170 and CAAE n^o^ 90426818.2.3001.5520 of July 6, 2018.

## RESULTS

The sample consisted of 461 individuals. The baseline clinical and demographic characteristics of the study participants are shown in Table 1. The average age was 62.8 (± 8.6) years and 77.4% were men. There was a high prevalence of cardiovascular risk factors: diabetes mellitus in 33.2%, dyslipidemia in 98.5%, hypertension in 88%, and current smoking in 33.4%. Most patients had normal left ventricular systolic function and 17.1% reported previous myocardial infarction. Regarding the anatomical characteristics of the coronary circulation, 83% of the cases had lesions in three or more epicardial vessels, with almost 30% showing an obstructive lesion in the left main coronary artery.

**Table 1:** Baseline characteristics of the sample.

Variables	N=461*
Age in years, mean (SD)	62.8 (8.6)
Male gender, % (n)	77.4 (357)
BMI in Kg/m^2^, mean (SD)	26.5 (4.1)
Smoking, % (n/N)	33.4 (149/446)
Diabetes, % (n/N)	37.2 (171/460)
SAH, % (n/N)	87.9 (394/448)
Dyslipidemia, % (n/N)	98.5 (393/398)
PAD, % (n/N)	4.7 (21/446)
COPD, % (n/N)	2.0 (9/450)
LV ejection fraction, % (n/N)	(N=437)
< 30%	0.4 (2)
30-50%	18.8 (82)
> 50%	80.8 (353)
MI < 90 days, % (n/N)	17.1 (77/450)
Prior PCI, % (n/N)	15.7 (72/458)
Myocardial revascularization, % (n/N)	1.5 (7/461)
Coronary lesions, % (n/N)	(N=461)
Uniarterial	3.7 (17)
Biarterial	12.0 (59)
Triarterial	51.0 (235)
Multiarterial (> 3)	32.5 (150)
LMC stenosis (> 50%), % (n/N)	28.7 (132/461)
Emergency surgery, % (n/N)	5.9 (27/458)
Preoperative creatinine, mg/dL - median (IIQ)	0.90 (0.8-1.1)
Preoperative creatinine clearance, ml/min - median (IIQ)	82.0 (66-102)
Preoperative risk scores:	
1-InsCor, median (IIQ)	6.0 (5.0-8.0)
2-EuroSCORE, median (IIQ)	1.24 (0.95-1.67)
3-STS mortality, median (IIQ)	0.68 (0.40-1.14)
4-STS mortality, median (IIQ)	9.24 (6.0-13.0)

BMI=body mass index; COPD=chronic obstructive pulmonary disease; EuroSCORE=European System for Cardiac Operative Risk Evaluation; IIQ=interquartile range; LMC=left main coronary; LV=left ventricular; MI=myocardial infarction; PAD=peripheral arterial disease; PCI=percutaneous coronary angioplasty; SAH=systemic arterial hypertension; SD=standard deviation; STS=Society of Thoracic Surgeons

* The number of observations showed differences according to the analyzed variable

Patients had preoperative risk assessed using mortality scores in cardiac surgery, with InsCor having a median of 6.0 (5.0-8.0); the EuroSCORE, a median of 1.24 (0.95-1.67); and the STS score a median of 0.68 (0.40-1.14) in the mortality variant and a median of 9.24 (6.0-13.0) in the morbidity variant. The intra and postoperative characteristics of the sample are shown in Table 2. Arterial grafts (internal mammary or radial arteries) were used in 72.5% of the cases and revascularization was complete in 53.4% of the patients (*i.e*., addressed all preoperative planned stenosis). The median of total in-hospital stay was eight days (7-10 days). Adverse postoperative outcomes were rare: myocardial infarction (2.8%), stroke (3.3%), and mediastinitis (0.22%); need for surgical reintervention occurred in only 1.7% of cases. Mortality in 30 days was 2.6% (12 cases).

**Table 2:** Intraoperative and postoperative characteristics of the sample.

Variables	N*=461
Coronary grafts, % (n/N)	
Arterial	72.5 (332/458)
Venous, bypass surgery	
1	26.5 (122/460)
2	53.4 (246/460)
3	16.0 (74/460)
4	1.1 (4/460)
Complete revascularization, % (n/N)	53.4 (246/460)
Use of blood components, % (n/N)	56.6 (254/449)
Hemodynamic instability, % (n/N)	2.4 (11/458)
Use of vasoactive agents, % (n/N)	
Ionotropic	50.7 (229/451)
Vasoconstrictor	20.1 (91/452)
Vasodilator	20.6 (93/451)
Blood dyscrasia, % (n/N)	21.0 (97/461)
Surgery time in minutes - median (IIQ)	210 (185-230)
CPB time in minutes - median (IIQ)	80 (65-95)
Intubation time in hours - median (IIQ)	7.5 (5.0-10.9)
Prolonged intubation (> 48 h), % (n/N)	1.7 (8/461)
ICU time in days - median (IIQ)	2 (2-2.25)
Total length of stay in days - median (IIQ)	8 (7-10)
Postoperative creatinine clearance, ml/min - median (IIQ)	92 (73-117)
Atrial fibrillation, % (n/N)	13 (60/461)
Stroke, % (n)	3.3 (15/456)
Acute MI, % (n)	2.8 (13/461)
Respiratory infection, % (n)	3.3 (15/456)
Mediastinitis, % (n)	0.22 (1/460)
Sepsis, % (n)	1.9 (9/461)
Cardiac tamponade, % (n)	0.87 (4/461)
Hemodialysis, % (n)	1.3 (6/461)
Reintervention for hemostasis revision, % (n)	1.7 (8/461)
Death, % (n)	2.6 (12/461)

CPB=cardiopulmonary bypass; ICU=intensive care unit; IIQ=interquartile range; MI=myocardial infarction

* The number of observations showed differences according to the analyzed variable

### InsCor, EuroSCORE, and STS Risk Scores

The risk scores were used to subdivide the population into three categories: low, medium, and high risk, according to pre-established criteria for each one. Table 3 shows the number of deaths in the study population by risk group for each model. InsCor had a higher mortality associated with a higher predicted risk. One death occurred in the low-risk group, two deaths in the medium-risk group, and nine deaths in the high-risk group. The STS group had deaths only in the group classified as having low risk, which represented the vast majority of the sample (94%). EuroSCORE performed almost similarly to STS. There were no high-risk patients in the STS and EuroSCORE scores in the studied sample.

**Table 3:** Incidence of death according to risk categories of preoperative scores.

Scores	N by risk level	Death, n (%)
InsCor		
Low risk (0-3)	88	1 (1.1)
Medium risk (4-7)	210	2 (1.0)
High risk (8)	163	9 (5.5)
STS*		
Low risk (0-2)	430	12 (2.8)
Medium risk (3-5)	29	0 (0)
EuroSCORE*		
Low risk (0-2)	379	10 (2.6)
Medium risk (3-5)	77	2 (2.6)

EuroSCORE=European System for Cardiac Operative Risk Evaluation; STS=Society of Thoracic Surgeons

* There were no patients within high-risk category (STS and EuroSCORE > 5).

### Discrimination Analysis of Risk Scores for Mortality Up to 30 Days

**Fig. 1:**
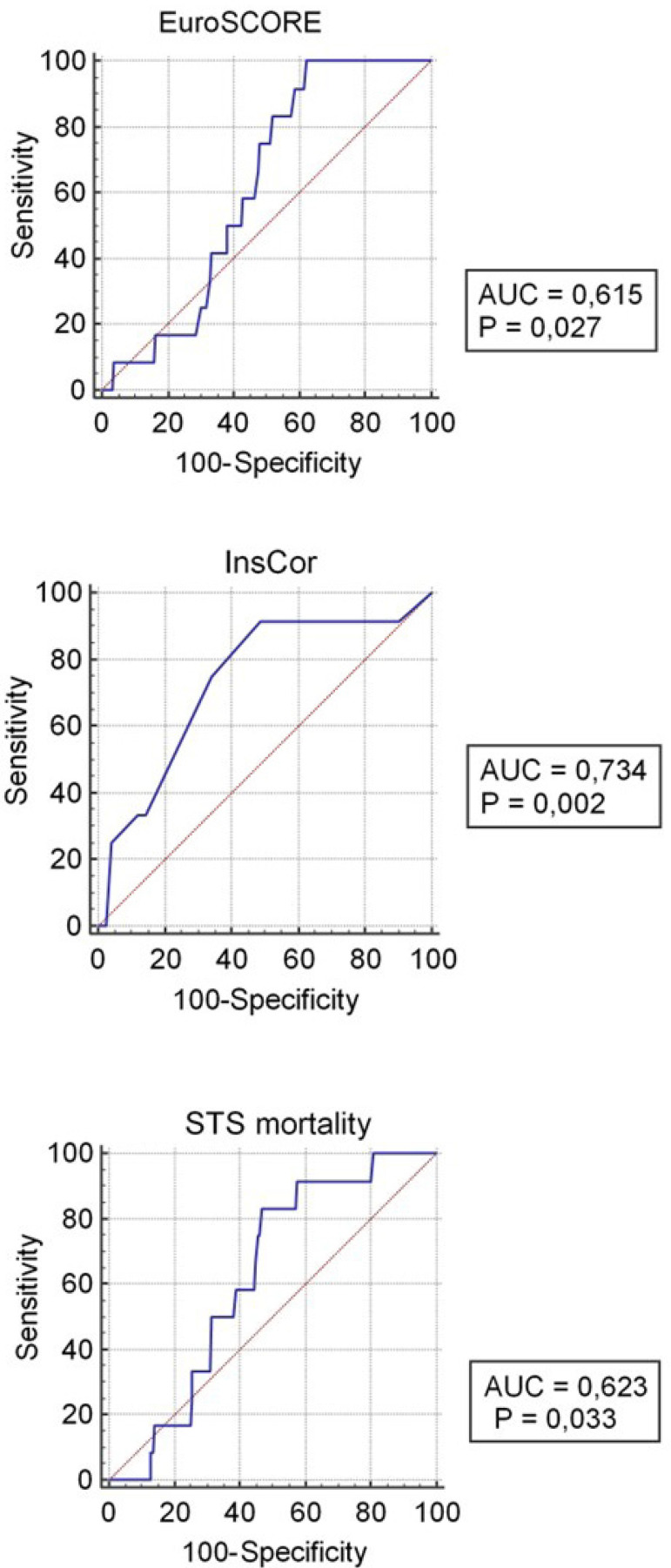
Receiver operating characteristic (ROC) curves for the studied mortality prediction scores. A) European System for Cardiac Operative Risk Evaluation (EuroSCORE) (area under the ROC curve [AUC]=0.615; P=0.025); B) InsCor (AUC=0.734; P=0.002); and C) Society of Thoracic Surgeons (STS) mortality (AUC=0.623; P=0.033). For all, the curve was drawn up containing sensitivity on the “y” axis and 100-specificity on the “x” axis.

Through calculations of the risk prediction scores for each patient in the study sample, ROC curves were created to assess the discrimination capacity of each risk model in relation to mortality up to 30 days after surgical myocardial revascularization. The EuroSCORE presented an area under the ROC curve of 0.615 (95% confidence interval [CI] [0.57-0.66], *P*=0.027); the STS score, an area of 0.623 (95% CI [0.58-0.69]; *P*= 0.033); and the InsCor score showed an area of 0.734 (95% CI [0.69-0.77]; *P*=0.002). InsCor had the best performance in C statistics when compared to the other scores evaluated. The three scores showed a statistically significant capacity for discrimination in the studied sample (*P*<0.05). However, the analysis of values of the areas under the ROC curve demonstrated that the EuroSCORE and STS scores showed modest capacities, while InsCor performed well. The calibration was obtained by comparing the expected mortality and observed by the Hosmer-Lemeshow test, proving to be adequate for InsCor (*P*=0.11), STS (*P*=0.19), and EuroSCORE (*P*=0.14) (Figure 1). The multivariate analysis including the three scores showed that InsCor score maintained a statistically significant association between higher levels of predicted risk and incidence of early death after CABG, after adjusting for EuroSCORE and STS. For the other two scores, the adjusted association did not reach statistical significance (Table 4).

**Table 4:** Multivariate analysis* comparing the performance of the InsCor, STS, and EuroSCORE risk scores for the mortality outcome.

Variable	Coefficient	Odds ratio	*P*-value	95% CI
InsCor	0.27	1.32	0.0144	1.06-1.64
STS, mortality	-0.1	0.9	0.79	0.44-1.89
EuroSCORE	0.18	1.19	0.53	0.67-2.13

CI=confidence interval; EuroSCORE=European System for Cardiac Operative Risk Evaluation; STS=Society of Thoracic Surgeons *Non-conditional multivariate logistic regression model.

## DISCUSSION

The present study, which evaluated the performance of three preoperative risk scores in predicting mortality in patients undergoing myocardial revascularization surgery, showed that the InsCor, STS, and EuroSCORE scores had good discrimination capacity, with InsCor being the model with better performance for predicting mortality within 30 days. In adjusted analysis between them, InsCor was the only model that maintained a statistically significant association for death. Mortality up to 30 postoperative days was 2.6%, which is in line with recent statistics of cohort studies composed of patients undergoing cardiac surgery for myocardial revascularization ^[^^10^^]^. Even if there were fewer patients with complete revascularization than would be expected (53.4%), which was explained by the preference of surgical team to graft the most important areas for heart function (there was a high percentage of patients with compromised medium to small coronaries and with a high calcification index), we believe that it would not have a significant impact in overall mortality, since the functional revascularization rate was high. Besides that, the frequency of using arterial grafts was also lower than expected in the present sample, but it represents the average statistics over six years of data collection. The use of arterial grafts certainly increased in the last years of the study and, currently, it occurs in a frequency > 95% of the patients submitted to CABG at the service.

Patients who undergo cardiac surgery are usually evaluated for their surgical risk, and some predictive models have been used in recent decades. EuroSCORE was developed in 1999 by Nashef et al. ^[^^11^^]^ and included 17 independent risk factors that were extracted from 19,030 patients who underwent isolated myocardial revascularization surgery (65%) or associated with valve surgery or interatrial communication, in 128 hospitals of eight European countries ^[^^12^^]^. In the derivation and validation cohorts ^[^^11^^]^, the model proved to be well calibrated and with reasonable discrimination capacity for in-hospital death (area under ROC curve of 0.76), whose incidence was 4.8%. In our sample, the performance of the EuroSCORE was lower (area under ROC curve of 0.615) than that reported in the original study, which can be explained by the methodological differences between the studies, especially regarding the sample size (higher in European cohorts), as well as the inclusion of patients undergoing procedures combined with myocardial revascularization in the original EuroSCORE studies. Diversities in the target populations from which the samples were obtained may also justify the heterogeneous results observed.

The STS score was developed in 1997 through an original assessment of 332,304 participants ^[^^9^^]^. In 2008, the score was revised to adapt to advances in cardiac surgery and improve its accuracy. The results of this review were published separately, according to the type of procedure performed: isolated myocardial revascularization, isolated valve surgery, or mixed cardiac procedures ^[^^13^^-^^15^^]^. In the first study, which included only isolated myocardial revascularization procedures as in ours, the calibration of the model was considered adequate, and the area under the ROC curve for postoperative mortality was 0.812, demonstrating good accuracy ^[^^13^^]^. This study evaluated more than 770 thousand patients in 819 centers, and in-hospital mortality was 2.3%. Although mortality was similar to that observed in the present study, the performance of the STS was lower in our sample (area under the ROC curve of 0.623). Very different size samples and divergences in the prevalence of risk factors for postoperative outcomes may justify the differences in results. However, it is noteworthy that risk models composed of many variables of different natures, as occurs in the STS score, tend to hinder their own interpretation, since elements related to patient care are mixed with their own preoperative clinical condition ^[^^4^^]^.

In an attempt to make a model more appropriate to Brazil’s reality and simpler for the daily application of the surgeons, the InsCor score was developed in 2012 at the Hospital da Universidade de São Paulo ^[^^4^^]^. Prospective data from 3,000 patients who underwent myocardial revascularization and/or valve surgery from 2007 to 2009 were analyzed. The sample had clinical characteristics similar to those described for the cohorts of other models, and mortality in the subgroup undergoing only myocardial revascularization surgery (1,641 patients) was 5.5%. The InsCor was completed with 10 variables and, in a validation cohort, it was shown to have an adequate calibration and regular to good discrimination power (area under the ROC curve of 0.79) ^[^^4^^]^. This finding was very similar to the one demonstrated in the present study, which may signal, as expected, a better consistency of InsCor when applied to Brazilian samples, even if they come from different regions of the country.

Few studies have compared InsCor directly with other models or compared the international scores themselves. The results of these studies, in turn, have many divergences and inconsistencies. In the InsCor development study, the national score was compared to the EuroSCORE, obtaining good calibration and discrimination (area under the ROC curve of 0.81), and it was superior to the European model in the same population ^[^^4^^]^. Subsequently, Lisboa et al. ^[^^16^^]^ compared EuroSCORE II with EuroSCORE and InsCor with respect to in-hospital mortality in 1,000 patients who underwent CABG and/or valve replacement surgery, between 2008 and 2009, at the Instituto do Coração/Universidade de São Paulo. In this study, the EuroSCORE and InsCor models had adequate calibration, which was not achieved by EuroSCORE II. On the other hand, the discrimination was adequate for the three scores: 0.81 (95% CI 0.76-0.85; P<0.001) for EuroSCORE II; 0.81 (95% CI 0.77-0.86; P<0.001) for EuroSCORE; and 0.79 (95% CI 0.74-0.83; P<0.001) for InsCor. Even though InsCor has presented slightly lower accuracy than the other scores, the study by Lisboa et al. brought a positive result in relation to the national score since the statistics were similar, and InsCor would have the advantage of being considerably more practical, with simplified formulas without the need for digital assistants at the bedside, potentially favoring their application ^[^^16^^]^. On the other hand, simpler scores tend to have good calibration and lower discrimination ^[^^17^^-^^19^^]^.

In 2004, Nilsson et al. ^[^^20^^]^, in Sweden, compared the accuracy of EuroSCORE with STS score for mortality up to 30 days, evaluating 4,497 patients who underwent isolated myocardial revascularization surgery, finding a mortality of 1.89%, adequate calibration in both models, and area under the ROC curve significantly greater for the EuroSCORE (0.84; 95% CI 0.80-0.88) than for the STS score (0.71; 95% CI 0.66-0.77), indicating, thus, a better performance of the European model. The results differ from those found in our cohort, where worse performance was detected for both models in relation to the national score. In addition to differences in sample size and population diversity, EuroSCORE and STS score did not identify high-risk patients in the present study, which may have influenced, in part, the results. It is reported, however, that the accuracy of the EuroSCORE tends to be lower for the highest risk subgroup.

The present work has many strengths. The first is the possibility of comparing InsCor with international scores in a cohort with a significant number of patients, operated in a Brazilian cardiology reference center, and with systematic follow-up up to 30 days after the operation. It is necessary to expand local assessments in order to be able to infer correctly about the performance of these risk models when applied to our population. On the other hand, it was possible to observe an apparent consistency in the performance of InsCor in the few cohorts where it was tested, showing it to be comparable to the other scores evaluated ^[^^4^^,^^16^^]^. An evident advantage of the InsCor score is the easiness of its application. This can be useful both to support its use and to point out nonconformities. Additionally, the opportunity to explore the behavior of the EuroSCORE and STS in a segment of the national population was important, with our study suggesting a poorer performance than those obtained from their original populations.

### Limitations

This study also has limitations. Although the data were collected prospectively, its analysis was retrospective, which brings bias susceptibility inherent to the study design. There is a potential selection bias, since patients with EuroSCORE and STS score at higher levels, absent in the sample, may have been contraindicated for myocardial revascularization surgery and underwent some alternative treatment (such as percutaneous coronary revascularization). In addition, the sample, although expressive, is small to truly assess the performance of InsCor, in addition to this being a single-center study. Our study evaluated EuroSCORE instead of EuroSCORE II, the revised model and, in theory, more accurate ^[^^8^^]^. However, there is inconsistency regarding the possible superiority of EuroSCORE II over the original model. Problems with the selection of EuroSCORE II’s derivation and validation cohorts, in addition to questions about their calibration, were raised ^[^^21^^]^. In addition, the newer European score has several related risk factors that may have led to an unsatisfactory calibration. There was also a large number of missing data in the follow-up, which may have altered the calculation of its coefficients ^[^^16^^]^. A study conducted in Turkey, for example, evaluated the EuroSCORE, EuroSCORE II, and STS scores in patients operated for isolated myocardial revascularization and demonstrated that EuroSCORE II had slightly better accuracy when compared to its previous version, but significantly underestimated the occurrence of death (EuroSCORE II predicted 1.7% and observed mortality was 7.9%) ^[^^22^^]^. Finally, the performance of the scores is related to the quality of services provided to the patient. Thus, the present study, like other studies that evaluate the performance mortality predictor scores, may lack external validity when applied to smaller services.

## CONCLUSION

The present study concluded that the InsCor score had a good predictive accuracy for death within 30 days of the postoperative period for patients undergoing CABG, with apparent superiority over the EuroSCORE and STS scores in our sample. However, further studies are needed, especially with larger samples and including high-risk patients, to validate the model and support its application.

**Table:** 

Authors' roles & responsibilities	
IFF	Substantial contributions to the conception or design of the work; or the acquisition, analysis, or interpretation of data for the work; drafting the work or revising it critically for important intellectual content; agreement to be accountable for all aspects of the work in ensuring that questions related to the accuracy or integrity of any part of the work are appropriately investigated and resolved; final approval of the version to be published
NAMR	Substantial contributions to the conception or design of the work; or the acquisition, analysis, or interpretation of data for the work; drafting the work or revising it critically for important intellectual content; agreement to be accountable for all aspects of the work in ensuring that questions related to the accuracy or integrity of any part of the work are appropriately investigated and resolved; final approval of the version to be published
VJCV	Substantial contributions to the conception or design of the work; or the acquisition, analysis, or interpretation of data for the work; drafting the work or revising it critically for important intellectual content; agreement to be accountable for all aspects of the work in ensuring that questions related to the accuracy or integrity of any part of the work are appropriately investigated and resolved; final approval of the version to be published
ALL	Substantial contributions to the conception or design of the work; or the acquisition, analysis, or interpretation of data for the work; drafting the work or revising it critically for important intellectual content; agreement to be accountable for all aspects of the work in ensuring that questions related to the accuracy or integrity of any part of the work are appropriately investigated and resolved; final approval of the version to be published
